# Asymmetric Ruthenium(II) and Osmium(II) Complexes with New Bidentate Polyquinoline Ligands. Synthesis and NMR Characterization

**DOI:** 10.3390/molecules15031324

**Published:** 2010-03-05

**Authors:** Antonino Mamo, Alessandro Aureliano, Antonino Recca

**Affiliations:** Dipartimento Metodologie Fisiche e Chimiche per l’Ingegneria, Facoltà di Ingegneria, Università di Catania, Viale A. Doria 6, 95125, Catania, Italy; E-Mail: alessandro.aureliano@gmail.com (A.A.)

**Keywords:** bidentate quinoline aza-ligands, ruthenium asymmetric complexes, osmium asymmetric complexes

## Abstract

A series of Ru(II) and Os(II) tris-chelate complexes with new bidentate 2-pyridylquinoline ligands have been synthesized and fully characterized by EA,^1^H-NMR and FAB-MS techniques. The new ligands are: **L_1_ =** 4-*p*-methoxyphenyl-6-bromo-2-(2′-pyridyl)quinoline (**mphbr-pq)** and **L_2_** = 4-*p*-hydroxyphenyl-6-bromo-2-(2′-pyridyl)- quinoline (**hphbr-pq)**. The complexes studied are: [Ru(bpy)_2_**L_1_**](PF_6_)_2_ (**C_1_**), [Ru(bpy)_2_**L_2_**](PF_6_)_2_ (**C_2_**), [Os(bpy)_2_**L_1_**](PF_6_)_2_ (**C_3_**), [Os(bpy)_2_**L_2_**](PF_6_)_2_ (**C_4_**) (bpy = 2,2′-bipyridine), [Ru(dmbpy)_2_**L_1_**](PF_6_)_2_ (**C_5_**), [Ru(dmbpy)_2_**L_2_**](PF_6_)_2_ (**C_6_**), [Os(dmbpy)_2_**L_1_**](PF_6_)_2_ (**C_7_**), and [Os(dmbpy)_2_**L_2_**](PF_6_)_2_ (**C_8_**) (dmbpy = 4,4′-dimethyl-2,2′-bipyridine). Moreover, new functionalized complexes **C_9_-C_12_** were obtained by the base-catalyzed direct alkylation of **C_2_**, **C_4_**, **C_6_**, and **C_8_** with 6-bromo-1-hexene. The complete assignment of the ^1^H-NMR spectra for the two new ligands (**L_1_** and **L_2_**), and their Ru(II) or Os(II) complexes has been accomplished using a combination of one- and two-dimensional NMR techniques. The *J*_H,H_ values have been determined for the majority of the resonances.

## 1. Introduction

Transition metal complexes have tremendous potential as diagnostic and therapeutic agents. They can be exploited for their modularity, reactivity, imaging capabilities, redox chemistry, and precisely defined three-dimensional structure [1,2,3,4,5]. Several [Ru(bpy)_3_]-derived (bpy = 2,2′-bipyridine) complexes were synthesized and compared electrochemically and spectroscopically in the search for better luminophores for electrochemiluminescence (ECL)-based analytical applications [6].

Electrochemiluminescence is a kind of sensitive process releasing light during reaction, which has potential applications in biological, pharmaceutical, and chemical land environmental analysis, due to its continuance, sensitivity, low-detection limit, reproducibility and relative easiness to be automatically controlled [7]. However, it is noted that different configurations in metal complexes affect a variety of activities, and most attention has been focused on symmetric aromatic ligands. On the other hand, only a limited number of ruthenium complexes containing asymmetric ligands have so far been described, and little attention has been paid to the investigation of their DNA-binding properties [8].

Among the factors governing the binding modes, it appears that the most significant is likely to be that of molecular shape. Studies reveal that the ligand modifications in geometry, size, hydrophobicity, planarity, and hydrogen-bonding ability of the complexes, may lead to suitable or spectacular changes in the binding modes, location, affinity, and to a different cleavage effect [9]. Therefore, inspired by Vos studies [10], we became interested in the synthesis of new polypyridyl ruthenium(II)/osmium(II) complexes with asymmetric ligands, with the aim of investigating (i) the effect of asymmetry on the photophysical properties of such compounds; (ii) their interaction properties with DNA [11]; (iii) their potential for the fabrication of new monolayers on both silica and Si substrates, namely to transfer molecular properties to the solid state thus obtaining photoluminescent devices [12,13].

**Ligand****R_1_****Nomenclature****Initials****L_1_**–OCH_3_*4-p-methoxyphenyl-6-bromo-2-(2′-pyridyl)quinoline***mphbr-pq****L_2_**–OH*4-p-hydroxyphenyl-6-bromo-2-(2′-pyridyl)quinoline***hphbr-pq**

**Scheme 1:**
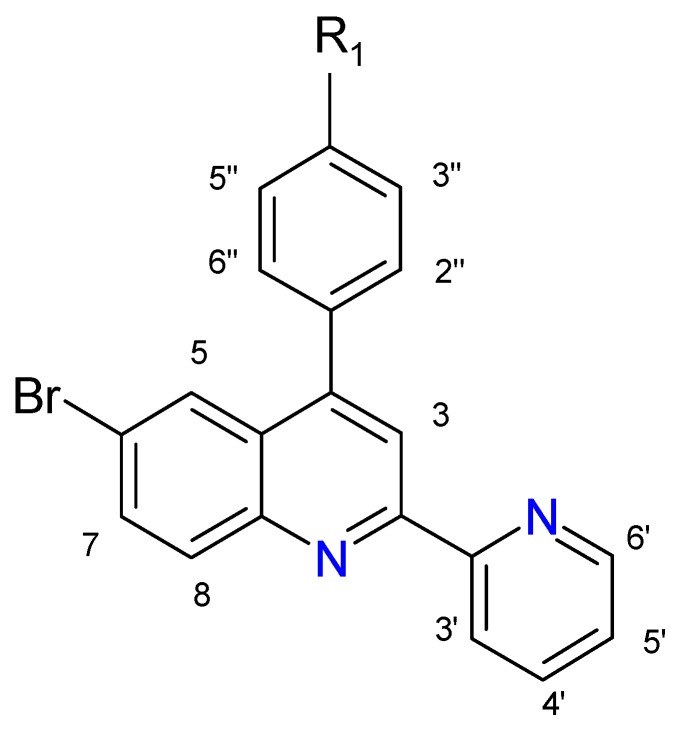
Ligand moiety.

**Scheme 2:**
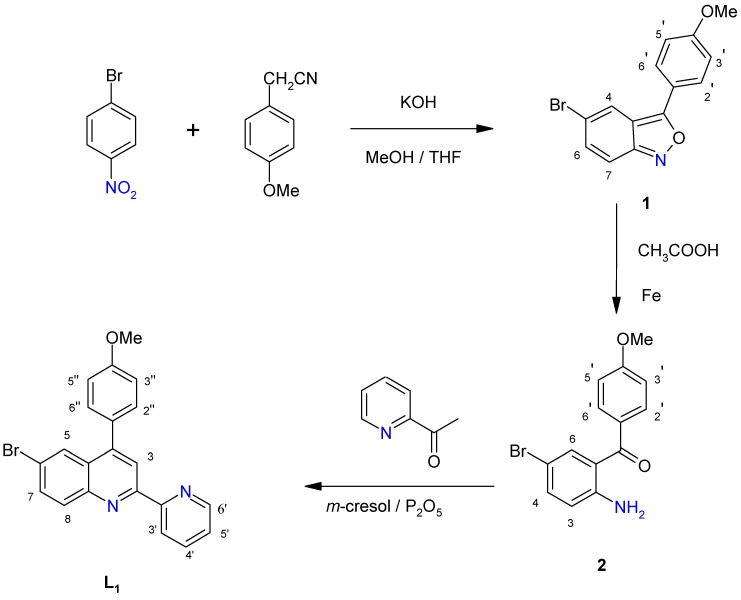
Synthesis of **L_1_**.

By continuing our previous studies in this field [14,15,16,17], we have designed two new asymmetric ligands (**L_1_**, **L_2_**), shown in Scheme 1, from which twelve aza-bidentate complexes with transition metals like ruthenium or osmium have been derived (See Scheme 2 and Scheme 5). This paper reports, as a first step, the synthesis and characterization of all the new compounds, while preliminary data on their luminescent properties, DNA binding, and solid state photoluminescent devices [18] will be reported elsewhere. All the compounds were characterized by EA, EI/FAB-MS and ^1^H-NMR spectroscopy. Complete assignments of the ^1^H spectra of the various compounds were accomplished by using a combination of one- and two-dimensional NMR techniques.

## 2. Result and Discussion

### 2.1. Ligands

Following the synthetic pathway previously used for the preparation of the parent ligand 4-phenyl-2-(2′-pyridyl)quinoline (**ph**-**pq**) [19], namely the acid-catalyzed condensation of *o-*amino- benzophenone with 2- acetylpyridine, we have synthesized ligand **L_1_** in a three synthetic steps as shown in Scheme 2. 

**Scheme 3:**
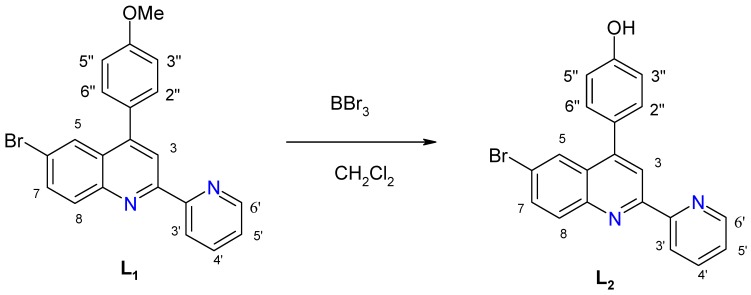
Synthesis of **L_2_**.

4’-Methoxy-2-amino-5-bromobenzophenone (**2**) was obtained by condensation of *p*-nitrobromo-benzene with *p*-methoxyphenylacetonitrile in a basic ethanol/tetrahydrofuran medium to give 3-*p*-methoxyphenyl-5-bromo-2,1-benzisoxazole (**1**) (66%), which upon reductive cleavage (Fe/CH_3_COOH) of the benzoisoxazole ring was converted to the desired aminoketone **2** (70%). A subsequent Friedlander reaction [20,21] of the *o-*aminobenzophenone **2** with 2-acetylpyridine, using a mixture of *m*-cresol and phosphorous pentoxide gave ligand **L_1_** (71%). The subsequent demethylation of **L_1_** with boron tribromide [22] afforded a new ligand 4-*p*-hydroxyphenyl-6-bromo-2-(2’-pyridyl)-quinoline (**brhph-pq**) (**L_2_**) as is shown in Scheme 3. 

The ^1^H-NMR spectroscopy proved to be a useful tool to check the structure of the synthesized ligands. By taking advantage of our previous ^1^H-NMR studies on similar compounds [16], we were able to make by comparison the overall proton assignments for our ligands that are reported in Table 1.

**Figure 1:**
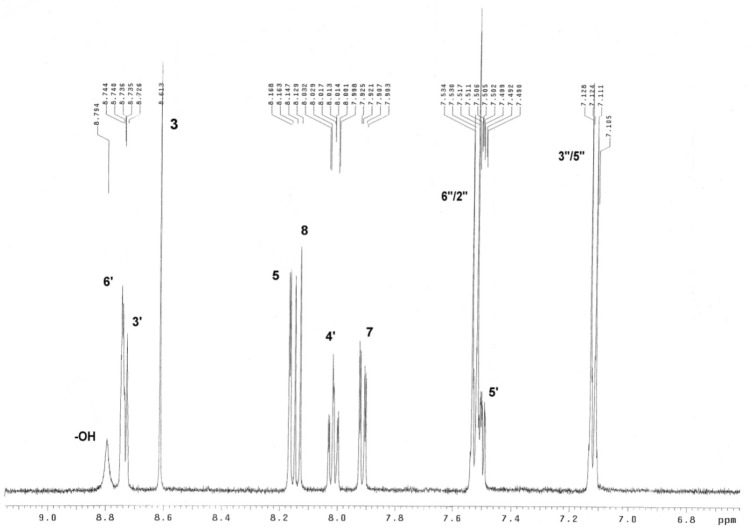
The ^1^H-NMR spectrum (500 MHz, (CD_3_)_2_CO) and peak assignments of ligand **L_2_**.

The ^1^H-NMR spectra of the synthesized ligands show the same trend in the region 7.0–9.0 ppm and in Figure 1 is reported an expanded section of the ^1^H-NMR spectrum in (CD_3_)_2_CO of **L_2_**, showing the assignments of all peaks as gathered in Table 1. In all cases, the spectra were found to be consistent with the expected structures. The ^1^H-NMR spectrum (Figure 1) of **L_2_** shows the expected pattern for a 2,4′′,6-trisubstituted 4-phenylquinoline moiety. In fact the two doublets at 8.60 and 8.73 ppm and two double triplets at 7.89 and 7.38 ppm, assigned to H^3^′, H^6^′, H^4^′ and H^5^′, respectively, is of diagnostic value for the presence of an α-pyridinyl group linked to the quinoline unit. Furthermore, one *AA*′*XX*′ system (two doublets at 7.52 and 7.09 ppm) –easily recognized because of its symmetry and apparent simplicity– accounts for a 1,4-disubstituted benzene ring having the 4′′ position occupied by the hydroxyl group (broad resonance at 8.79 ppm). Finally, the lack of any signal for a hypothetic H^6^ proton along with the multiplicity of H^8^, H^7^ and H^5^ protons is in agreement with the presence of the bromine substituent, as confirmed by elemental analysis.

### 2.2. Complexes

**Scheme 4:**
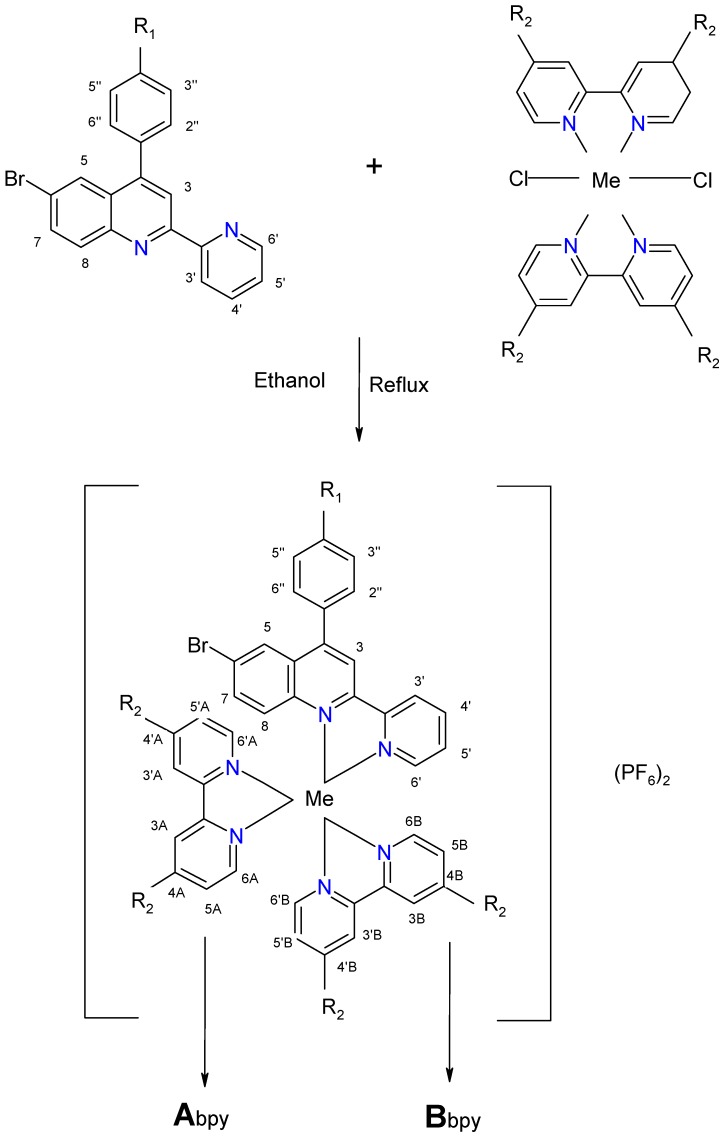
Synthesis of complexes **C_1_**–**C_8_**.

According to Scheme 4, by crossing the ligands **L_1_** or **L_2_** with the starting *cis* form of bis-chelate Ru(bpy)_2_Cl_2_, Os(bpy)_2_Cl_2_, Ru(dmpy)_2_Cl_2_, and Os(dmbpy)_2_Cl_2_ complexes where the two bpy or dmbpy (dmbpy = 4,4′-dimethyl-2,2′bipyridine) units lie on orthogonal planes and the chlorine atoms occupy adjacent coordination sites, we have synthesized eight new tris-chelate complexes **C_1_–C_8_**.

**Complex****Ligand****Me****R_1_****R_2_****Chemical Formula****C_1_****L_1_**Ru-OCH_3_H[Ru(bpy)_2_L_1_] (PF_6_)_2_**C_2_****L_2_**Ru-OHH[Ru(bpy)_2_L_2_] (PF_6_)_2_**C_3_****L_1_**Os-OCH_3_H[Os(bpy)_2_L_1_] (PF_6_)_2_**C_4_****L_2_**Os-OHH[Os(bpy)_2_L_2_] (PF_6_)_2_**C_5_****L_1_**Ru-OCH_3_CH_3_[Ru(dmbpy)_2_L_1_] (PF_6_)_2_**C_6_****L_2_**Ru-OHCH_3_[Ru(dmbpy)_2_L_2_] (PF_6_)_2_**C_7_****L_1_**Os-OCH_3_CH_3_[Os(dmbpy)_2_L_1_] (PF_6_)_2_**C_8_****L_2_**Os-OHCH_3_[Os(dmbpy)_2_L_2_] (PF_6_)_2_

**Scheme 5:**
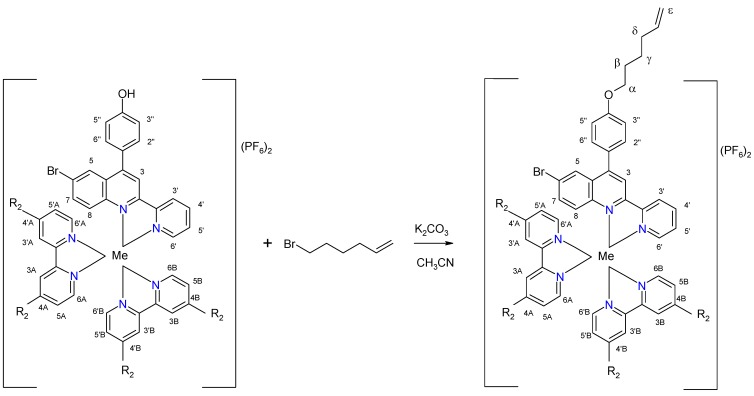
Synthesis of complexes **C_9_**–**C_12_**.

The syntheses were accomplished by reacting equimolar amounts of the reagents in refluxing ethanol for 8 h, followed by dropwise addition of a 20% water solution of NH_4_PF_6_, in order to get the red-orange complexes. These were collected by filtration and purified by crystallization. Owing to the ability of alkene molecules to covalently bond to hydrogen-terminated crystalline silicon (111) by thermally induced hydrosilylation [23], the required *cis* octahedral coordinated complexes **C_9_–C_12_** were prepared by direct alkylation of **C_2_**, **C_4_**, **C_6_**, and **C_8_**, respectively, with 6-bromo-1-hexene in K_2_CO_3_/CH_3_CN mixture, as shown in Scheme 5. These new tris-chelate complexes exhibit, besides the two bpy or dmbpy moieties, the new *4-p-(5-hexen-1-yloxy)phenyl-6-bromo-2-(2*′*-pyridyl)quinoline* ligand (**L_3_**).

**Complex****Ligand****Me****R_1_****R_2_****Chemical Formula****C_9_**L_3_RuO(CH_2_)_4_CH=CH_2_H[Ru(bpy)_2_L_3_](PF_6_)_2_**C_10_**L_3_OsO(CH_2_)_4_CH=CHH[Os(bpy)_2_L_3_](PF_6_)_2_**C_11_**L_3_RuO(CH_2_)_4_CH=CHCH_3_[Ru(dmbpy)_2_L_3_](PF_6_)_2_**C_12_**L_3_OsO(CH_2_)_4_CH=CHCH_3_[Os(dmbpy)_2_L_3_](PF_6_)_2_

The synthesized complexes were generally stable, diamagnetic, and kinetically inert. The ^1^H-NMR spectra of Ru(bpy)_2_Cl_2_ and Os(bpy)_2_Cl_2_ show [24,25] eight different signals for the aromatic hydrogens, that become six for Ru(dmbpy)_2_Cl_2_, and Os(dmbpy)_2_Cl_2_, consistently with the presence in solution of two non-interconverting enantiomers possessing *C_2_* symmetry. Substitution of the two chlorine atoms with ligands **mphbr-pq** (**L_1_**) or **hphbr-pq** (**L_2_**) yields the corresponding tris-chelate complexes which, in a *cis* octahedral coordination, are also capable of existing in two enantiomeric propeller conformations, but, in contrast to the above cited bis-chelate complexes, do not possess a *C_2_* axis of symmetry.

The reduced symmetry of these complexes removes the degeneracies associated with the *C_2_* axis in the starting Ru(bpy)_2_Cl_2_, giving rise to a structure of the general type [Me(bpy)_2_L](PF_6_)_2_, (being Me = Ru or Os) and L is an unsymmetrical bidentate ligand like **L_1_** or **L_2_**. As a consequence, howing to the asymmetry of the complexes, and to the kinetically restricted interconversion of the two enantiomers on the NMR time-scale, the ^1^H-NMR spectra of complexes **C_1_–C_12_** are quite complicated showing 16 signals for the diastereomeric protons of the two bpy moieties (**A**bpy and **B**bpy) in addition to 13 signals for the ligand **L_1_** or **L_2_** and 18 for **L_3_**, as reported in Table 2.

**Figure 2:**
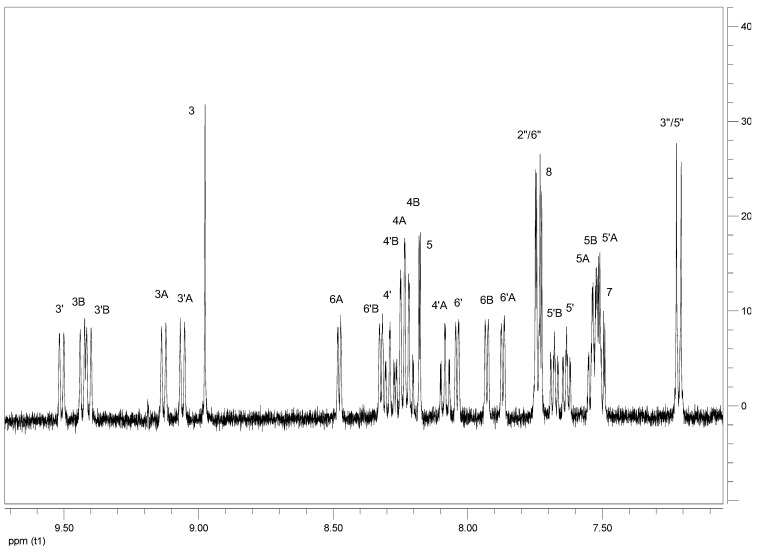
The ^1^H-NMR spectrum (500 MHz, (CD_3_)_2_CO) and peak assignments of complex **C_1_**.

**Figure 3:**
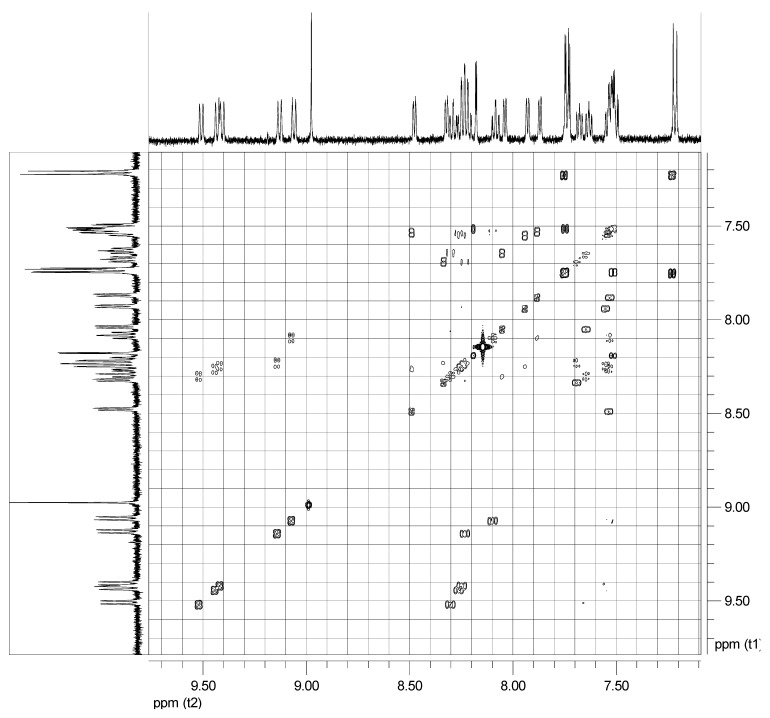
^1^H/^1^H COSY-45 spectrum (500 MHz, (CD_3_)_2_CO) of **C_1_**.

As an example, Figure 2 shows the downfield aromatic section of the ^1^H NMR spectrum of complex **C_1_**. The twentyeight methine resonances, arising from the two bipyridyl units (**A**bpy and **B**bpy) and to the bidentate ligand (**mphbr-pq**) (**L_1_**) are spread out over a 2.5 ppm interval. As described in the following, each signal has been assigned to the respective proton with the aid of mono and two-dimensional techniques. Chemical shifts and assignments are reported in Table 2 and Figure 3. 

The assignment of the sole singlet in the spectrum (8.98 ppm, H^3^) is straightforward. In fact, it is worth to note that the 3-positioned aromatic proton of the pyridine moiety in Ru(II) complexes displays a remarkable deshielding of ca 0.45 ppm, as compared to the free ligands, that is considered diagnostic for their formation.

In the COSY-45 spectrum of **C_1_** (see Figure 3) correlations between *ortho*, *meta* and *para* protons are normally observable; differentiation among ^1^*J*, ^2^*J* and ^3^*J* couplings has been greatly aided by the careful examination of shape and intensity of the cross-peaks. Starting from the peak at 9.51 (H^3′^), the sequence of signals at 8.29 (H^4′^), 7.63 (H^5′^) and 8.04 ppm (H^6′^) can be assigned; another set of connectivities (signals at 8.18/7.50/7.74 ppm) allows to assign the sequence H^5^, H^7^, and H^8^ of the ligand **mphbr-pq**. Unambiguous identification of H^8^ (7.74) is based on the upfield shift observed for this proton in 1H NMR spectrum of the Ru(II) complex **C_1_** with respect to the free ligand **L_1_** (see Table 1), probably due to the shielding effect of a pyridine ring approximately orthogonal to this proton; H^5^ shows, on the contrary, a negligible downfield shift. The intense peaks at 7.73 and 7.22 ppm indicate the presence of an *AA*^′^*XX*^′^ system diagnostic of *p*-disubstituted benzene ring, and may be easily attributed to the resonances of four phenyl protons of **mphbr-pq**.

Following inspection of the COSY spectrum, further sequences of isolated four-spin systems can be analyzed. Using low-field signals as convenient starting points, it is possible to establish the complete set of connectivities for the following sequences of signals: 9.43/8.22/8.54/7.93 ppm, 9.41/8.23/7.68/8.32 ppm, 9.13/8.24/7.54/8.48 ppm and 9.06/8.08/7.52/7.87 ppm. Owing to a combination of inductive and steric effects, the proton H^7^, H^8^, and H^6’^ of the asymmetric ligands experience, upon complexation, an upfield effect with respect to the free ligands as is shown in Table 2. It is also worth noting that in the case of complexes with **dmbpy** moieties (**C_5_**–**C_8_**) we observe two different signals for the four methyl groups in the ratio 1:3 (with the less intense signal experiencing an upfield effect), suggestive of the presence of a clear steric effect between the big bromo substituent linked to the quinoline moiety of the asymmetric ligands and the methyl group linked to the nearest pyridine ring of the **A**bpy moiety. (See Scheme 4)

On the basis of this evidence, after inspection of the molecular models and taking into account the resonances of H^6^ and/or H^6′^ bpy protons, we assume that binding of the asymmetric ligands (**L_1_** or **L_2_**) is expected to strongly shield these protons and shift them to higher field. In other words, upon complexation the nearest is the proton of the bpy units to the asymmetric ligand, the stronger is the upfield effect experienced. As a consequence, in accord with the numbering pattern shown in Scheme 4 and/or Scheme 5, resonances at 7.87, 7.93, 8.32,and 8.48 ppm were assigned to H^6′A^, H^6B^, H^6′B^ and H^6A^, respectively. Therefore the former pair of sequence signals is assigned to the protons H^3B^ and H^3’B^ of the same bpy unit, respectively, and the latter one to H^3A^ and H^3′A^ of the other bpy unit. The assignments of the above cited signals to the sequences from position 3 to position 6 of the bpy ligands are reported in Table 2.

## 3. Experimental

### 3.1. General

The starting materials, 2-acetylpyridine, 2-aminobenzophenone, *p*-nitrobromobenzene, *p*-methoxyphenylacetonitrile, 4,4’-dimethylbpy, and 6-bromo-1-hexene, were purchased from Aldrich. All other chemicals were reagent grade. Os(bpy)_2_Cl_2,_ Ru(dmbpy)_2_CL_2_ and Os(dmbpy)_2_CL_2_ were synthesized by the method of Togano *et al.* for Ru(bpy)_2_Cl_2_ and were used without purification [26]. All reactions were performed under an inert atmosphere of nitrogen except when otherwise stated and the solvents were dried and stored under nitrogen and over 4Å molecular sieves. Melting points are uncorrected. Elemental analyses were determined commercially. The analytical and FAB-MS data of complexes **C_1_**–**C_12_** are gathered in Table 3. Proton spectra were performed in (CD_3_)_2_CO or CDCl_3_ by using a Varian INOVA 500 MHz instrument. ^1^H-NMR spectra were calibrated relative to the solvent resonance considered at 2.05 or 7.26 ppm for residual (CH_3_)_2_CO or CHCl_3_, respectively. The analysis of the proton spectra was carried out according to the rules for the first-order splitting with the help of integral intensities, and resonance splitting patterns are abbreviated by using s for singlet, d for doublet, dd for doublet of doublets, t for triplet, and m for multiplet. Positive ion FAB mass spectra were obtained on a Kratos MS 50 S double-focusing mass spectrometer equipped with a standard FAB source, using 3-nitrobenzyl alcohol as the matrix. The ^1^H spectra with assigned signals are given in Table 2. 

### 3.2. Syntheses

*3-p-Methoxyphenyl-5-bromo-2,1-benzoisoxazole* (**1**). To a vigorously stirred solution containing potassium hydroxide (17.76 g, 310 mmol) in methanol (35 mL) at room temperature, was slowly added *p*-methoxyphenylacetonitrile (1.75 g, 15 mmol). After dissolution was complete, a methanol/tetrahydrofuran (2:1, v/v) solution (36 mL) containing *p*-nitrobromobenzene (3.0 g, 15 mmol) was added dropwise at 0 °C. The resulting dark mixture was stirred at 0 °C for 3 h, at room temperature for 4 h, refluxed overnight, and then poured into ice-water (300 mL) to afford, after filtration, cold water and methanol washings and methanol recrystallization, compound **1** as yellow crystals; 2.22 g (66%); m.p. 112 °C; ^1^H-NMR (CDCl_3_) δ: 8.05 (bs, 1H, H^4^ of benzoisoxazole); 7.94 (d, 2H, *J* = 8.5 Hz, H^2’^/H^6’^of phenyl), 7.50 (dd, 1H, *J* = 9.5, 1.0 Hz, H^7^ of benzoisoxazole); 7.36 (dd, 1H, *J* = 9.5, 1.5 Hz, H^6^ of benzoisoxazole); 7.09 (d, 2H, *J* = 8.5 H^3’^/H^5’^of phenyl), 3.92 (s, OCH_3_); MS, *m/z* 304 (MH^+^); Anal. Calcd. for C_14_H_10_BrNO: C, 56.95; H, 2.92; N, 5.11. Found: C, 57.19; H, 3.03; N, 4.86. 

*4’-Methoxy-2-amino-5-bromobenzophenone* (**2**). A solution containing **1** (0.44 g, 1.6 mmol) in acetic acid (70 mL), was heated on a water-bath, and iron powder (1.0 g, 18 mmol) was added over 2.5 h, during which time water (12 mL) was also added. The mixture was filtered while hot and then water (100 mL) was added. The yellow precipitate was collected by filtration, washed with cold water until the water washings were clear and dried. The product was purified by column chromatography (silica; cyclohexane/ethyl acetate 9:1) followed by recrystallization from ethanol-water to afford **2** as a yellow powder; 031 g (70%); m.p. 105 °C; ^1^H-NMR (CDCl_3_) δ: 7.68 (d, 2H, *J* = 9.0 Hz, H^2’^/H^6’^of phenyl); 7.55 (d, 1H, H^6^ of benzene); 7.35 (dd, 1H, *J* = 8.5, 2.5 Hz, H^4^of benzene); 6.98 (d, 2H, *J* = 9.0 Hz, H^3’^/H^5’^of phenyl); 6.65 (d, 1H, *J* = 9.0 H^3^ of benzene) 5.83 (bs, 2H, of amino) 3.90 (s, CH_3_ of methoxy); MS, *m/z* 406 (MH^+^); Anal. Calcd. for C_14_H_11_BrNO: C, 56.54; H, 3.62; N, 5.07. Found: C, 56.28; H, 3.59; N, 4.95

*4-p-Ethoxyphenyl-6-bromo-2-(2’-pyridyl)quinoline* **(brmph-pq, L_1_)**. A mixture of *m*-cresol (25 mL) and phosphorus pentoxide (0.81 g, 5.7 mmol) was stirred at 145 °C for 2.5 hours to afford a homogeneous solution. After cooling, 4-methoxy-2-amino-5-bromobenzophenone (4.08 g, 15 mmol) and 2-acetylpyridine (2.03 g, 15 mmol) were added, followed by *m*-cresol (20 mL) to rinse the powder funnel. The reaction mixture was heated at 135 °C overnight. After cooling, the dark solution was poured into ethanol (200 mL) containing triethylamine (20 mL). The light grey precipitate was collected by filtration, continuosly extracted with a solution of ethanol/triethylamine for 24 h, and recrystallized from *n*-hexane/methylene chloride to give **brmph-pq (L_1_)** as an off white powder; 3.96 g (71%); m.p. = 212 °C.; MS, *m/z* 375 (MH+).

*4-p-Hydroxyphenyl-6-bromo-2-(2’-pyridyl)quinoline* (**brhph-pq, L_2_**). A mixture of of **L1** (0.5 g, 1.27 mmol), 1 M boron tribromide in dichloromethane ((5.69 mL, 5.08 mmol) and dichloromethane (dry, 30 mL) was stirred at −75 °C for 0.5 h and room temperature for 24 h. The reaction mixture was poured into ice and cold water (800 mL) and stirred for 0.5 h. The red precipitate was filtrated and suspended in ethanol (250 mL). The turbid red mixture was neutralized by some drops of 1N NaOH (colour changing from red to light-blue). The light-blue precipitate was collected by filtration, washed with cold water and ethanol until the water washings were clear and dried under vacuum on P_2_O_5_ at 40 °C to give 0.42 g (72%) of **L_2_** as a white solid. 

The synthesis of complex **C_1_** is given below as a general procedure for the synthesis of **C_1_-C**_8_ complexes.

*[Ru(bpy)_2_(brmph-pq)] (PF_6_)_2_*. (**C_1_**). To a refluxing solution of *cis*-Ru(bpy)_2_Cl_2_ 2H_2_O (0.156 g, 0.3 mmol) in ethanol (20 mL), was added dropwise a solution of **brmph-pq** (0.1 g, 0.35 mmol) in EtOH (20 mL), and the mixture was allowed to reflux for 8 h. After concentration and addition of water (15 mL), the mixture was refluxed for 5 min and filtered while hot. After cooling, the complex was precipitated by dropwise addition of a 20% water solution of NH_4_PF_6_ (5 mL). The red precipitate was collected, washed with cold water and Et_2_O, and purified by gel filtration on a column of Sephadex LH-20 in EtOH followed by recrystallization from acetone-Et_2_O, to give 0.22 g (72%) of **C_1_** as red orange crystals.

The synthesis of complex **C_9_** is given below as a general procedure for the synthesis of **C_9_–C**_12_ complexes. 

*[Ru(bpy)_2_(**L_2_**-hexene)](PF_6_)_2_*. To a refluxing solution of K_2_CO_3_ (0,2 g, 14.5 mmol) in CH_3_CN (20 mL) was added dropwise a solution of [Ru(bpy)_2_(**brhph-pq**)](PF_6_)_2_ (0.06g. 0.055 mmol) in CH_3_CN (10 mL) and a solution of 6-bromo-1-hexene (0.018 g. 0.111 mmol) in CH_3_CN (10 mL) and the mixture was allowed to reflux for 48 h. Then the reaction mixture was filtered while hot to remove carbonate-salt, rotoevaporated to dryness, dissolved with a minumum quantity of acetone and poured into Et_2_O. The red-brown precipitate was collected by filtration, washed with Et_2_O, and purified by recrystallization from acetone-Et_2_O, to give 0.06 g (99%) of **C_9_** as dark-red crystals.

## 4. Conclusions

A series of Ru(II) and Os(II) complexes with new polyquinoline asymmetric aza-bidentate ligands have been synthesized and characterized by EA, EI-FAB Mass and NMR techniques. Complete ^1^H NMR assignments have been obtained by the use of two-dimensional techniques. The results indicate that steric hindrance on Ru(II) or Os(II) metal has to be carefully considered in designing new bidentate asymmetric ligands. Because of their intrinsic asymmetry, the racemic complexes obtained, when resolved into their enantiomeric forms, will provide interesting species for DNA binding studies, the development of solid state photoluminescent devices, light harvesting compounds, and useful energy traps when inserted into supramolecular arrays.

## Figures and Tables

**Table 1:** ^1^H-NMR spectral data for ligands **L_1_** and **L_2_**. Coupling constants in italic.

Proton	3	5	7	8	3′	4′	5′	6′	2′′/6′′	3′′/5′′	OCH_3_	OH
**L_1_**	8.51 s	8.13 d *J* = 2.0	7.80 dd *J* = 9.0 *J* = 2.0	8.10 d *J* = 8.0	8.60 d *J* = 8.0	7.89 dt *J* = 8.0 *J* = 1.5	7.38 dt *J* = 9.0 *J* = 1.5	8.73 d *J* = 4.0	7.52 d *J* = 9.0	7.09 d *J* = 9.0	3.92 s	-
**L_2_**	8.61 s	8.16 d *J* = 2.5	7.91dd *J* = 9.0, *J* = 2.0	8.14 d *J* = 9.0	8.73 d *J* = 8.0	8.01 dt *J* = 8.0, *J* = 1.5	7.50 dt *J* = 8.5, *J* = 1.5	8.74 d *J* = 4.0	7.53 d *J* = 9.0	7.12 d *J* = 9.0	-	8.79 bs

Notes: The spectra were obtained in (CD_3_)_2_CO, chemical shifts in ppm, and coupling constants (*J*) in Hz. Numbering pattern as shown in Scheme 3. Abbreviations used: s = singlet, d = doublet, dd = double doublet, t = triplet, dt = double triplet, bs = broad singlet, bd = broad doublet.

**Table 2:** ^1^H-NMR spectral data for complexes **C_1_**–**C_12_**. Coupling constants in italic.

	C_1_	C_2_	C_3_	C_4_	C_5_	C_6_	C_7_	C_8_	C_9_	C_10_		C_11_	C_12_
**H^3^**	8.98s	8.79s	8.94s	8.75s	9.01s	8.76s	8.97s	8.58s	8.81s	8.80s		8.84s	8.83s
**H^5^**	8.18d *J* = 2.0	8.23d *J* = 2.0	8.12d *J* = 2.0	8.17d *J* = 2.0	8.18d *J* = 2.0	8.21d *J* = 2.0	8.12d *J* = 2.0	8.10d *J* = 2.0	8.18d *J* = 2.5	8.15d *J* = 2.0		8.18d *J* = 2.0	8.15d*J* = 2.0
**H^7^**	7.50 dd *J* = 9.0, 2.0	7.50dd *J* = 9.0,2.0	7.46dd *J* = 9.0,2.0	7.46dd *J* = 8.5,2.0	7.49dd *J* = 9.0,2.0	7.50m	7.45dd *J* = 9.0, 2.0	7.50m	7.50dd *J* = 9.0,2.0	7.45dd *J* = 8.5,2.0		7.49dd *J* = 9.0,2.0	7.46dd *J* = 9.0,2.0
**H^8^**	7.74d *J* = 9.0	7.73d *J* = 9.5	7.69d *J* = 9.0	7.68d *J* = 9.0	7.82d *J* = 9.0	8.11d *J* = 9.0	7.77d *J* = 9.0	7.97d *J* = 9.0	7.73d *J* = 9.5	7.67d*J* = 9.0		8.10d *J* = 9.5	7.84d *J* = 9.0
**H^3′^**	9.51d *J* = 8.5	9.23d *J* = 9.0	9.46d *J* = 8.5	9.16d *J* = 8.0	9.57d *J* = 9.0	9.18d *J* = 8.5	9.52d *J* = 9.0	9.18d *J* = 8.5	9.23d *J* = 9.0	9.36d *J* = 8.5		9.29d *J* = 8.5	9.42d *J* = 9.0
**H^4′^**	8.29 dt *J* = 8.0, 1.5	8.32m	8.07m	8.10m	8.23dt *J* = 8.0, 1.5	8.27dt *J* = 8.0,2.0	8.01m	8.18m	8.32dd *J* = 8.02.0	8.07t*J* = 8.0		8.26t *J* = 8.0	8.01t *J* = 8.0
**H^5′^**	7.63dt *J* = 7.0,1.5	7.65t *J* = 7.5,1.5	7.61dt *J* = 7.5,1.5	7.63m	7.62dt *J* = 7.0,1.5	7.60t *J* = 8.0	7.60dt *J* = 7.0,1.5	7.62t *J* = 8.0	7.62m	7.62m		7.61t *J* = 7.0	7.61t *J* = 7.0
**H^6′^**	8.04d *J* = 5.0	8.06d *J* = 5.5	8.04d *J* = 5.0	8.06d *J* = 5.0	8.02d *J* = 5.0	8.02d *J* = 5.5	8.02d *J* = 5.0	7.96d *J* = 5.0	8.05d *J* = 5.0	7.88d*J* = 5.0		8.03d *J* = 5.5	7.86d *J* = 5.0
**H^2”/6”^**	7.73d *J* = 9.0	7.55d *J* = 8.5	7.72d *J* = 9.0	7.54d *J* = 8.5	7.71d *J* = 8.5	7.55d *J* = 8.0	7.70d *J* = 8.5	7.53d *J* = 8.5	7.70d *J* = 8.5	7.54d*J* = 8.5		7.68d *J* = 8.5	7.52d *J* = 8.5
**H^3′′/5′′^**	7.22d *J* = 9.0	7.14d *J* = 8.5	7.20d *J* = 9.0	7.12d *J* = 8.5	7.21d *J* = 8.5	7.13d *J* = 8.0	7.19d *J* = 8.5	7.14d *J* = 8.5	7.23d *J* = 9.0	7.15d *J* = 8.5		7.22d *J* = 8.5	7.14d *J* = 8.5
**H^3A^**	9.13d *J* = 8.0	8.76d *J* = 8.0	9.08d *J* = 8.0	8.71d *J* = 8.5	9.28bs	8.62bs	9.23bs	8.60bs	8.77d *J* = 8.0	8.98d *J* = 8.5		8.92bs	9.13bs
**H^4A^**	8.24m	8.26m	8.03m	8.05m					8.24dd *J* = 8.02.0	8.05m			
**H^5A^**	7.53m	7.58m	7.43m	7.48m	7.48m	7.50m	7.38m	7.44m	7.55m	7.46m		7.50m	7.41m
**H^6A^**	8.48d *J* = 5.0	8.52d *J* = 6.0	8.35d *J* = 5.0	8.39d *J* = 5.5	8.22bd	7.75bd	8.09m	7.78m	8.50d *J* = 6.0	8.38d *J* = 5.5		7.76d *J* = 5.5	7.64d *J* = 5.0
**H^3′A^**	9.06d *J* = 8.0	8.68d *J* = 8.0	8.99d *J* = 8.0	8.61d *J* = 8.0	9.19bs	8.53bs	9.12bs	8.48bs	8.69d *J* = 9.0	8.87d *J* = 8.0		8.83bs	9.01bs
**H^4′A^**	8.08 dt *J* = 8.0, 1.0	8.11t *J* = 7.5	7.89t *J* = 8.0	7.92t *J* = 8.0					8.11t *J* = 7.5	7.92t *J* = 8.0			
**H^5′A^**	7.52 m	7.56m	7.37 m	7.41m	7.31 m	7.32m	7.16 m	7.24 m	7.52m	7.44m		7.31m	7.23 m
**H^6′A^**	7.87d *J* = 5.0	7.91d *J* = 6.0	7.81d *J* = 5.5	7.85d *J* = 5.0	7.35d *J* = 5.0	7.38d *J* = 5.5	7.29d *J* = 5.5	7.42m	7.89d *J* = 5.5	7.76d *J* = 7.5		7.37d *J* = 6.0	7.24m
**H^3B^**	9.43d *J* = 8.0	8.96d *J* = 8.0	9.39d *J* = 8.0	8.92bs	9.61bs	8,78bs	9.57bs	8.74bs	8.97d *J* = 8.0		9.26d *J* = 8.0	9.15bs	9.44bs
**H^4B^**	8.23 m	8.28m	8.02 m	8.07m					8.27 dt *J* = 8.01.5		8.07m		
**H^5B^**	7.53 m	7.57m	7.30 m	7.44m	7.31 m	7.34m	7.18 m	7.30 m	7.54m		7.43m	7.32m	7.21 m
**H^6B^**	7.93d *J* = 5.0	7.99d *J* = 5.0	7.84d *J* = 5.0	7.90bd	7.66d *J* = 5.0	7.73d *J* = 5.0	7.67bd	8.16bd	7.97d *J* = 5.5		7.85d *J* = 5.0	7.70d *J* = 6.0	7.58d *J* = 5.5
**H^3′B^**	9.41d *J* = 8.0	8.93d *J* = 7.5	9.39d *J* = 7.5	8.91bd	9.60bs	8.75bs	9.56bs	8.70bs	8.94d *J* = 8.5		9.24bd	9.13bs	9.43bs
**H^4′B^**	8.23m	8.30m	8.04m	8.09m					8.28 dd *J* = 8.01.5		8.09m		
**H^5′B^**	7.68dt *J* = 7.0, 1.5	7.70t *J* = 7.0	7.64dt *J* = 7.0, 1.5	7.66m	7.64d *J* = 7.0	7.65d *J* = 7.5	7.60d *J* = 7.0	7.36m	7.68t *J* = 7.5		7.66t *J* = 7.5	7.64d *J* = 7.5	7.62d *J* = 7.0
**H^6′B^**	8.32d *J* = 6.0	8.36d *J* = 5.0	8.16d *J* = 6.0	8.20d *J* = 5.5	8.22d *J* = 6.0	8.23d *J* = 6.5	8.06d *J* = 6.0	7.70d *J* = 6.0	8.34d *J* = 5.5		8.21d *J* = 6.5	8.20d *J* = 6.0	7.62d *J* = 6.0
**CH_3_^′^A**					2.49bs	2.45bs	2.44bs	2.50bs				2.48bs	2.47bs
**CH_3_B**					2.60bs	2.54bs	2.53bs	2.70bs				2.59bs	2.53bs
**CH_3_A**					2.60bs	2.55bs	2.53bs	2.71bs				2.60bs	2.53bs
**CH_3_^′^B**					2.60bs	2.56bs	2.53bs	2.72bs				2.61bs	2.53bs
**αCH_2_**									4.17 t *J* = 6.5		4.17 t *J* = 6.5	4.17 t *J* = 6.5	4.17 t *J* = 6.5
**βCH_2_**									1.87t *J* = 6.5		1.86t *J* = 6.5	1.87t *J* = 6.5	1.87t *J* = 6.5
**γCH_2_**									1.63t *J* = 6.5		1.62t*J* = 6.5	1.63t *J* = 6.5	1.63t *J* = 6.5
**δCH_2_**									2.17dt *J* = 7.0, 1.5		2.15dt *J* = 7.0, 1.5	2.17dt *J* = 7.0, 1.5	2.16dt *J* = 7.0, 1.5
**εCH_2_**									5.00dd *J* = 10.0, 2.0		5.04dd *J* = 10.0, 2.0	5.02dd *J* = 10.0, 2.0	5.00dd *J* = 10.0, 2.0
**-CH**									5.87m		5.88m	5.87m	5.87m
**OCH_3_**	3.95s		3.95s		3.95s		3.95s						
**-OH**		9.95bs		9.96bs		9.95bs		9.96bs					

Notes: The spectra were obtained in deuterated acetone (CD_3_)_2_CO, chemical shifts in ppm, and coupling constants (*J*) in Hz. Numbering pattern as shown in Scheme 4 and Scheme 5. Abbreviations used: s = singlet, d = doublet, dd = double doublet, t = triplet, dt = double triplet, bs = broad singlet, bd = broad doublet.

**Table 3:** Analytical and Positive ion FAB-MS Spectral Data for the Complexes **C_1_**–**C_12_**.

Complex.	Yield (%) (XXX) Me_2_CO/Et_2_O	Molecular Formula (M.W.)	%C Found (Calcd)	%H Found (Calcd)	%N Found (Calcd)	FAB–MS*m/z*
**C_1_**	72	Ru(C_41_H_31_BrF_12_N_6_OP_2_) (1094.50)	44.96 (45.00)	3.00 (2.85)	7.56 (7.67)	949 [Ru(bpy)_2_**L_1_**](PF_6_)^+^
**C_2_**	63	Ru(C_40_H_29_BrF_12_N_6_OP_2_) (1080.47)	44.33 (44.46)	2.85 (2.70)	7.47 (7.77)	935 [Ru(bpy)_2_**L_2_**](PF_6_)^+^
**C_3_**	69	Os(C_41_H_31_BrF_12_N_6_OP_2_) (1183.66)	42.01 (41.62)	2.22 (2.64)	7.37 (7.10)	1038 [Os(bpy)_2_**L_1_**](PF_6_)^+^
**C_4_**	71	Os(C_40_H_29_BrF_12_N_6_OP_2_) (1169.63)	41.74 (41.41)	2.09 (2.50)	7.33 (7.18)	1024 [Os(bpy)_2_**L_2_**](PF_6_)^+^
**C_5_**	95	Ru(C_45_H_39_BrF_12_N_6_OP_2_) (1150.60)	46.91 (46.97)	3.57 (3.40)	6.94 (7.30)	1005 [Ru(dmbpy)_2_**L_1_**](PF_6_)^+^
**C_6_**	80	Ru(C_44_H_37_BrF_12_N_6_OP_2_) (1136.57)	46.11 (46.49)	3.19 (3.28)	7.41 (7.39)	991 [Ru(dmbpy)_2_**L_2_**](PF_6_)^+^
**C_7_**	73	Os(C_45_H_39_BrF_12_N_6_OP_2_) (1239.76)	46.52 (46.13)	2.49 (2.90)	6.74 (6.78)	1094 [Os(dmbpy)_2_**L_1_**](PF_6_)^+^
**C_8_**	80	Os(C_44_H_37_BrF_12_N_6_OP_2_) (1225.73)	42.98 (43.11)	2.85 (3.04)	6.92 (6.89)	1080 [Os(dmbpy)_2_**L_2_**](PF_6_)^+^
**C_9_**	99	Ru(C_46_H_39_BrF_12_N_6_OP_2_) (1162.61)	47.63 (47.52)	3.49 (3.38)	6.93 (7.23)	1017 [Ru(bpy)_2_**L_3_**](PF_6_)^+^
**C_10_**	94	Os(C_46_H_39_BrF_12_N_6_OP_2_) (1251.77)	44.52 (44.13)	3.06 (3.14)	6.58 (6.71)	1106 [Ru(bpy)_2_**L_3_**](PF_6_)^+^
**C_11_**	83	Ru(C_50_H_47_BrF_12_N_6_OP_2_) (1218.65)	48.91 (49.27)	4.11 (3.89)	6.87 (6.89)	1099 [Ru(dmbpy)_2_**L_3_**](PF_6_)^+^
**C_12_**	88	Os(C_50_H_47_BrF_12_N_6_OP_2_) (1307.81)	46.09 (45.91)	3.36 (3.62)	6.80 (6.42)	1188 [Os(dmbpy)_2_**L_3_**](PF_6_)^+^
